# Clinical, immunological, molecular characteristics and outcomes of stem cell transplantation in *ZAP70* deficiency: a single-center experience

**DOI:** 10.3389/fimmu.2025.1656240

**Published:** 2025-09-25

**Authors:** Fai AlQahtani, Azhar Al Shaqaq, Bandar Al-Saud, Nora AlRumayyan, Reem Mohammed, Rand Arnaout, Sahar Elshorbagi, Sultan Albuhairi, Ali Al-Ahmari, Mouhab Ayas, Hawazen Al-Saedi, Abbas Hawwari, Anas M. Alazami, Hamoud Al-Mousa

**Affiliations:** ^1^ Department of Pediatrics, Collage of Medicine, King Fahad Hospital of the University, Alkhobar, Saudi Arabia; ^2^ Department of Pediatrics, King Faisal Specialist Hospital & Research Center, Riyadh, Saudi Arabia; ^3^ College of Medicine, Alfaisal University, Riyadh, Saudi Arabia; ^4^ Department of Pediatric Hematology/Oncology, King Faisal Specialist Hospital and Research Center, Riyadh, Saudi Arabia; ^5^ Department of Translational Genomics, Genomic Medicine Centre of Excellence, King Faisal Specialist Hospital & Research Center, Riyadh, Saudi Arabia

**Keywords:** *ZAP-70* deficiency, CD8 deficiency, severe combined immunodeficiency disease, SCID, hematopoietic stem cell transplant, immunodeficiency, inborn error of immunity

## Abstract

**Background:**

Zeta-chain-associated protein kinase 70 (*ZAP70*) deficiency is a rare autosomal recessive T^+^B^+^NK^+^ combined immunodeficiency characterized by heterogeneous clinical and immunologic phenotypes. Because of the limited number of reported cases, data guiding optimal management and hematopoietic stem cell transplantation (HSCT) strategies remain scarce.

**Methods:**

We retrospectively reviewed all patients with genetically confirmed *ZAP70* deficiency treated at King Faisal Specialist Hospital and Research Centre. Data on pre-HSCT clinical and immunologic features, transplant characteristics, post-HSCT complications, immune reconstitution, and long-term outcomes were recorded.

**Results:**

Thirteen patients with a median age at symptom onset of 1 month were identified. The most frequent initial presentations were recurrent respiratory infections and cutaneous manifestations. Autoimmune complications and lymphoproliferation were observed in several patients. Eleven of thirteen patients (84.6%) exhibited profound CD8^+^ T-cell lymphopenias and two had near-normal CD8^+^ T-cell counts with impaired T-cell function. Eleven patients underwent HSCT, including seven from Human Leukocyte Antigen (HLA)-matched family donors, two from one-antigen mismatched related donors, one from a haploidentical mother (matched for graft-versus-host disease risk but mismatched for rejection), and one from an unrelated cord blood donor. Two patients required a second transplant because of poor immune reconstitution. Of the 11 patients who underwent HSCT, 8 (73%) remain alive with a median follow-up of 7 years (range, 1–15), and most demonstrated resolution of clinical manifestations.

**Conclusion:**

HSCT remains the only curative treatment for *ZAP70* deficiency. Myeloablative conditioning regimens appear to promote more robust and durable immune reconstitution. In critically ill patients with severe infections or end-organ damage, reduced-intensity or unconditioned HSCT can be considered as a life-saving approach, although subsequent interventions might be necessary.

## Introduction

Severe combined immunodeficiency (SCID) comprises a life-threatening group of inborn errors of immunity characterized by a profound defect in T-cell differentiation, often accompanied by abnormalities in B and/or natural killer (NK) cell development and, more rarely, the myeloid lineage (1, 2). The clinical phenotype of SCID is typically consistent, being marked by early-onset severe and recurrent infections, interstitial pneumonitis, oral candidiasis, and chronic diarrhea and often accompanied by growth failure (3). SCID encompasses a genetically and clinically heterogeneous group of disorders, with more than 22 distinct genetic defects identified to date. These variants can be classified according to the cellular phenotype, inheritance pattern, and underlying gene defect (4–11). Several SCID-related genetic mutations involve disruptions in T-cell receptor (TCR) signaling. Upon antigen recognition, the TCR complex transmits intracellular signals via non-receptor cytoplasmic protein tyrosine kinases (PTKs), particularly Lck and ζ-chain-associated protein kinase of 70 kDa (ZAP70) (12).

ZAP70 is a cytoplasmic member of the Syk family of non-receptor PTKs, and it is predominantly expressed in T-cells, in which it plays pivotal roles in their development and activation (12, 13). Structurally, ZAP70 consists of two tandem SH2 domains and a carboxy-terminal kinase domain. Upon TCR engagement, the SH2 domains of ZAP70 bind to phosphorylated immunoreceptor tyrosine-based activation motifs on the TCR ζ-chain, recruiting ZAP70 to the activated complex (12). ZAP70 then phosphorylates key downstream molecules, including SLP-76 and LAT, thereby initiating signaling cascades essential for T-cell activation, differentiation, cytokine production, adhesion, and motility (14). Activated ZAP70 also contributes to the functional regulation of various lymphocyte subsets, including γδ T-cells, NK cells, MAIT cells, naïve and memory CD8^+^ T-cells, regulatory T-cells, and naïve CD4^+^ T-cells (12). Importantly, ZAP70 plays a central role in the positive selection of CD8^+^ thymocytes, whereas CD4^+^ T-cell development is relatively spared because of compensation by the related kinase Syk, which is more highly expressed in human thymocytes than in murine thymocytes (15). In *ZAP70*-deficient patients, thymic histology typically reveals CD4^+^CD8^+^ double-positive cells in the cortex but predominantly CD4^+^ single-positive cells in the medulla (16). The absence of ZAP70 protein thus results in selective CD8^+^ T-cell deficiency with impaired signal transduction in CD4^+^ T-cells in humans, whereas in murine models, complete *ZAP70* deficiency leads to an arrest in both CD4^+^ and CD8^+^ T-cell development because of insufficient Syk expression (17).

*ZAP70* deficiency was first described in 1994 as a form of SCID characterized by lymphocytosis with selective CD8^+^ T-cell deficiency and functional defects of CD4^+^ T-cells (18). *ZAP70* deficiency was initially identified in patients of Mennonite descent and subsequently detected in other ethnicities, including Hispanic, Japanese, Kurdish, Turkish, Portuguese, Caucasian, Mexican, Malagasy, and Iranian patients. Most patients had an initial diagnosis of SCID. Patients with *ZAP70* deficiency present with a spectrum of clinical manifestations, including recurrent respiratory tract infection, dermatitis, or chronic diarrhea, and they require hematopoietic stem cell transplantation (HSCT) to survive (13, 17). *ZAP70* deficiency is an exceptionally rare form of SCID, with only a limited number of cases reported to date. In this study, we described the clinical, immunologic, and genetic characteristics, as well as transplantation outcomes and post-HSCT immune reconstitution, of 13 previously unpublished patients diagnosed with *ZAP70* deficiency who were treated at a single tertiary care center.

## Methods

### Patients

This retrospective chart review involved all patients with genetically confirmed *ZAP70* deficiency diagnosed between 2000 and 2024 and followed at the Immunodeficiency Clinics at King Faisal Specialist Hospital and Research Centre (KFSHRC, Riyadh, Saudi Arabia). We evaluated the clinical, immunological, and genetic characteristics; HSCT details; post-transplant outcomes; and immune reconstitution of all patients at the most recent follow-uThe study was approved by the Institutional Review Board at KFSHRC (Reference Number: 2241016; Approval Date: January 25, 2024). Written informed consent was obtained from the patients or minor(s)’ legal guardian for the publication of any potentially identifiable images or data included in this article.

### Cellular and immunological assays

Peripheral blood leukocyte subsets were analyzed using immunofluorescence staining and flow cytometry. Monoclonal antibodies specific for T-cells (CD3, CD4, CD8), NK cells (CD16, CD56), and B cells (CD19) were used (Becton, Dickinson & Co., Franklin Lakes, NJ, USA) (19). T-cell functional capacity was assessed *in vitro* by evaluating proliferative responses to phytohemagglutinin (PHA) stimulation, as previously described (20). Serum immunoglobulin (Ig) levels, including IgG, IgA, and IgM levels, were measured by nephelometry (21).

### Sequencing of *ZAP70*

Genomic DNA was extracted from peripheral blood samples collected via venipuncture from patients and their parents as part of routine clinical practice. In earlier cases (2000–2016), Sanger sequencing of ZAP70 was performed based on clinical suspicion, using polymerase chain reaction amplification followed by direct sequencing, as previously described (21). From 2016 onward, next-generation sequencing (NGS) was employed, either through targeted primary immunodeficiency panels or whole-exome sequencing, with variant confirmation by Sanger sequencing and familial segregation analysis (22). Sequence reads were aligned to the reference human genome (GenBank), and variants were identified and analyzed. To confirm the novelty of identified variants, the Saudi Human Genome Database was consulted to exclude unreported population-specific single nucleotide polymorphisms. Additionally, in silico prediction tools were employed to assess the potential pathogenicity of identified missense variants.

### Preparative regimen, transplantation, and supportive care

Eleven patients underwent HSCT at a median age of 7 months (range, 3–84). HLA compatibility was assessed using high-resolution molecular typing at class I (HLA-A, HLA-B, HLA-C) and class II (HLA-DRB1, HLA-DQB1) loci. Stem cell sources included unmanipulated bone marrow from HLA-matched siblings (n = 3) and HLA-matched parents (n = 4), as well as one unrelated umbilical cord blood donor. Patient P9 received a graft from his father, and patient P10 received a graft from his brother, each with a single HLA antigen mismatch. Patient P4, who was homozygous at HLA loci, received a transplant from his mother, who was considered fully matched for graft-versus-host disease (GVHD) risk but haploidentical concerning rejection. Eight patients underwent HSCT without pre-transplant conditioning because of active infections at presentation, whereas three patients received conditioning regimens, including fludarabine, melphalan, and antithymocyte globulin (ATG) in P4 and P11; busulfan and cyclophosphamide in P10; and busulfan, fludarabine, and ATG in P8 for a second transplant following the failure of initial unconditioned HSCT. The number of infused CD34^+^ stem cells ranged from 3.75 × 10^6^ to 10 × 10^6^/kg in related donor transplants. In the umbilical cord blood transplant recipient (P11), 0.11 × 10^6^/kg CD34^+^ cells and 5.15 × 10^8^/kg total nucleated cells were infused. P4, whose first transplant was performed with reduced-intensity conditioning, received a stem cell boost 2 years after transplantation because of suboptimal immune reconstitution. GVHD prophylaxis was not used in patients who received unconditioned transplants from HLA-matched siblings, whereas cyclosporine (CSA) was used for unconditioned transplants from HLA-matched parents. In patients who received conditioning, GVHD prophylaxis included the calcineurin inhibitors CSA (P4, P9, P10, P11) and tacrolimus (P8), methotrexate (P8, P10), and mycophenolate mofetil (P9, P11), and corticosteroids were added for the unrelated cord blood transplant recipient (P11). Patients were housed in positive-pressure isolation rooms throughout the transplant period. Aplasia-associated febrile episodes were treated with empiric intravenous antibiotics. Hematological support included transfusions of irradiated, CMV-negative erythrocytes and platelets, as clinically indicated. Pre-emptive antiviral prophylaxis with intravenous acyclovir (1500 mg/m^2^/day for 60 days) was administered to CMV-negative recipients with CMV-positive donors. All patients received G-CSF (5 μg/kg/day) until the absolute neutrophil count exceeded 0.5 × 10^9^/L and intravenous immunoglobulin (IVIG) every 3 weeks. Chimerism was monitored using short tandem repeat typing. The diagnosis of acute and chronic GVHD followed established consensus criteria, with confirmation by histopathology when necessary. All patients received corticosteroids as first-line therapy, and additional immunomodulatory treatments were used as required.

### Immune reconstitution

Immune reconstitution was defined as a composite of clinical, cellular, functional, and humoral recovery. Clinical reconstitution included resolution of recurrent or severe infections, clearance of pre-existing viral infections (e.g., CMV, EBV), and the ability to discontinue antimicrobial prophylaxis. Cellular reconstitution was defined as normalization of CD3^+^ T-cell counts (>1,000/µL) with recovery of CD8 subset. Functional reconstitution was evidenced by restoration of T-cell proliferative responses to mitogen stimulation (≥10% of control). Humoral reconstitution was defined by independence from IVIG replacement, sustained normalization of serum immunoglobulin levels (IgG, IgA, and IgM), and demonstration of protective vaccine-specific antibody responses. Durable donor lymphoid chimerism was also considered supportive evidence of successful reconstitution.

## Results

### Patients’ characteristics and clinical presentations

Thirteen patients from nine unrelated families with genetically confirmed *ZAP70* deficiency were included in this study. All 13 patients were born to consanguineous parents, corresponding to a consanguinity rate of 100% in this cohort. The cohort comprised seven boys and six girls. A positive family history suggestive of primary immunodeficiency was documented in 12 patients (92.3%), and three patients were diagnosed through targeted newborn screening because of family history. The median age at diagnosis was 4 months (range, 1–84). All patients exhibited chronic dermatitis, most commonly with eczema-like features. Eleven patients (84.6%) had recurrent respiratory tract infections, whereas eight (61.5%) had chronic diarrhea. CMV infection occurred in five patients (38.5%), including two who developed CMV retinitis. Epstein–Barr virus (EBV) viremia was documented in two patients (15.4%). Bacille Calmette–Guérin (BCG) vaccine-related complications were detected in four patients (30.8%), including three with localized BCGitis and one with disseminated disease. Lymphadenopathy, with or without hepatosplenomegaly, was observed in five patients. Seven patients exhibited failure to thrive or poor weight gain, primarily attributed to chronic gastrointestinal symptoms. Two patients (15.4%) exhibited autoimmune manifestations in the form of autoimmune hemolytic anemia and nephrotic syndrome. The detailed clinical features of each patient are summarized in **Table 1**.

**Table 1:** Clinical, immunological and molecular characteristics of 13 patients with *ZAP-70* deficiency.

Patient/family code	Gender	Disease onset (months)	Age at diagnosis (months)	Clinical presentation	Immunological characteristics				*ZAP-70* homozygous variant (NM_001079.4)	HSCT
					CD3 mm^3^ (Normal range)	CD8 mm^3^ (Normal range)	PHA% (patient/control)	Igg g/L (normal range)		
P1, F1	Male	4	24	Recurrent chest infection, chronic dermatitis, chronic diarrhea, localized BCGitis, food allergy, FTT, left axillary LAP and splenomegaly, right liver lobe cyst	2516 (1800 – 4500)	66 (500 – 1500)	0	15.1 (4-12)	c.569G>C: p.R190T	Yes
P2, F2	Female	60	84	Recurrent chest infection, chronic dermatitis, AIHA, CMV and EBV viremia, and FTT	1510 (1200 – 2800)	836 (300 – 1100)	1	14.9 (6-15)	c.1606G>A: p.G536S	Yes
P3, F2	Female	72	72	Chronic dermatitis, chronic diarrhea, CMV and EBV viremia, FTT, allergic conjunctivitis, and food allergy	3140 (1500 – 3500)	593 (400 – 1200)	0	11.4 (5-14)	c.1606G>A: p.G536S	Yes
P4, F3	Male	1	1	Chronic dermatitis, chronic diarrhea, AIHA, CMV viremia and retinitis, bronchial asthma, and bilateral nephrocalcinosis	4905 (2000 – 6000)	62 (500 – 2000)	1	9.5 (2-9)	c.1606G>A: p.G536S	Yes
P5, F4	Male	1	4	Recurrent chest infection, Omenn syndrome, localized BCGitis, left axillary LAP, and FTT	3302 (2000 – 5000)	56 (500 – 1800)	ND	5.3 (2-10)	c.1606G>A: p.G536S	Yes
P6. F5	Male	2	7	Recurrent chest infection, chronic dermatitis, oral thrush, and laryngomalacia	2490 (2000 – 5000)	17 (500 – 1800)	ND	8.6 (3-12)	c.492delC: p.His165fs	Yes
P7, F4	Female	1	1	Recurrent chest infection, chronic dermatitis, chronic diarrhea, oral thrush, and simple splenic cyst	2618 (2000 – 6000)	80 (500 – 2000)	ND	5.5 (2-9)	c.1606G>A: p.G536S	Yes
P8, F4	Female	1	2	Recurrent chest infection, chronic dermatitis, FTT and Nephrotic syndrome	4673 (2000 – 6000)	92 (500 – 2000)	ND	4 (2-9)	c.1606G>A: p.G536S	Yes
P9, F6	Male	1	3	Recurrent chest infection, chronic dermatitis, chronic diarrhea, CMV viremia and retinitis, LAP, HSM, and small scalp hemangioma	536 (2000 – 6000)	71 (500 – 2000)	ND	4 (2-9)	c.1606G>A: p.G536S	Yes
P10, F6	Male	1	1	Recurrent chest infection, chronic dermatitis, and CMV viremia	4788 (2000 – 6000)	56 (500 – 2000)	ND	5.6 (2-9)	c.1606G>A: p.G536S	Yes
P11, F7	Female	1	19	Recurrent chest infection, chronic dermatitis, chronic diarrhea, disseminated BCGitis, with bilateral cervical, axillary and inguinal LAP	4251 (1800 – 4500)	0 (500 – 1500)	0	9.6 (4-12)	c.1082 + 2T>C	Yes
P12, F8	Female	1	1	Recurrent chest infection, chronic dermatitis, chronic diarrhea	1005 (2000 – 6000)	5 (500 – 2000)	0	8.4 (2-9)	c.1082 + 2T>C	No
P13, F9	Male	10	11	Recurrent chest infection, chronic dermatitis, chronic diarrhea, localized BCGitis, FTT, cervical LAP, Hepatomegaly	345 (2000 – 5000)	0 (500 – 1800)	ND	4.5 (3-12)	c.1332_1338delGGTGTCC: p.V445fs*3	No

HSCT, Hematopoietic Stem Cell Transplantation; BCGitis, Bacillus Calmette-Guérin Infection; FTT, failure to thrive; AIHA, Autoimmune hemolytic anemia; CMV, Cytomegalovirus; EBV, Epstein-Barr virus; LAP, lymphadenopathy; HSM, hepatosplenomegaly; PHA, phytohemagglutinin.

### Immunological characteristics

The median white blood cell count across the cohort was 11.0 × 10^9^/L, with a median absolute neutrophil count (ANC) of 2.79 × 10^9^/L and a median absolute lymphocyte count of 5.18 × 10^9^/L. CD8^+^ T-cell lymphopenia was a consistent diagnostic clue, being observed in 11 patients (84.6%), with CD8^+^ counts ranging 0–92 cells/mm^3^. Notably, two patients (P2 and P3) had normal CD8^+^ T-cell counts for age (median, 715.5 cells/mm^3^). The median counts for other lymphocyte subsets were as follows: CD3^+^ T-cells, 2618 cells/mm^3^; CD4^+^ T-cells, 2481 cells/mm^3^; CD19^+^ B cells, 636 cells/mm^3^; and CD56^+^/CD16^+^ NK cells, 449 cells/mm^3^. Lymphocyte proliferation assays in response to PHA stimulation were performed in six patients, and severely reduced responses (0%–1%) compared with those in healthy controls were identified. Serum Ig levels were generally within normal ranges for most patients. However, two patients exhibited low IgA levels, and one had decreased IgM levels. The patients’ detailed immunological profiles are summarized in **Table 1**.

### Molecular genetic testing

All thirteen patients were found to carry homozygous mutations in *ZAP70*. Eight patients (P2, P3, P4, P5, P7, P8, P9, and P10), originating from four different families within the same tribe, harbored a previously reported missense mutation: NM_001079.4: c.1606G>A: p.Gly536Ser. This mutation results in the replacement of a nonpolar, neutral glycine with a polar, neutral serine residue in the kinase domain. Patient P1 carried a homozygous missense mutation in exon 5 (c.569G>C; p.R190T), changing the amino acid from arginine (a basic amino acid) to threonine (a neutral amino acid), as previously described (22). Two patients from unrelated tribes (P11 and P12) carried the splice-site mutation c.1082 + 2T>C, which is predicted to disrupt normal splicing. Homozygous frameshift deletions were identified in two patients. P6 had c.492delC: p.His165fs, resulting in a histidine-to-threonine substitution followed by a frameshift, whereas P13 had c.1332_1338delGGTGTCC: p.Val445fs*3, leading to a premature stop codon three amino acids downstream. All variants were confirmed by Sanger sequencing, and familial segregation analysis verified that each mutation co-segregated with the disease phenotype in a pattern consistent with autosomal recessive inheritance. The patients’ detailed genetic findings are presented in **Table 1**.

### HSCT engraftment, survival, and toxicity

Eleven patients underwent a combined 13 HSCT procedures. The overall post-transplant survival rate was 72.7% (8/11) over a median follow-up duration of 7 years (range, 1–15). Three patients (P9, P10, P11) died from transplant-related complications, including septicemia in one patient (P9) and acute respiratory distress syndrome in two patients (P10, P11). Among patients who received conditioning regimens, the median time to neutrophil recovery (ANC > 0.5 × 10^9^/L) was +26 days, and that to platelet recovery (>20 × 10^9^/L without transfusion) was +23.5 days. Acute GVHD occurred in two patients. Specifically, P5 and P11 developed skin GVHD, and P11 additionally experienced liver and gastrointestinal involvement. All cases were biopsy-confirmed, and the patients responded to systemic corticosteroids. No chronic GVHD was observed. Infectious complications were frequent, affecting 63.6% (7/11) of patients. These included CMV viremia, EBV viremia, and septicemia. Patient P4, who received reduced-intensity conditioning, experienced poor immune reconstitution with persistent CMV viremia and autoimmune hemolytic anemia (AIHA). A subsequent stem cell boost led to immune recovery and CMV clearance. AIHA was controlled in this patient with rituximab and IVIG, which he continues to receive. Patient P8, who underwent her first transplant without conditioning, developed AIHA and steroid-resistant nephrotic syndrome associated with glomerulonephritis, considered signs of graft failure or inadequate immune reconstitution. She subsequently received a second transplant from the same donor using a myeloablative conditioning regimen, resulting in full donor chimerism (100%) and the complete resolution of autoimmunity. She remains off all immunosuppressive and supportive therapy 1 year post-transplant. Patients who received transplants without conditioning or with reduced-intensity conditioning displayed partial donor lymphoid chimerism (38%–94%) but no donor myeloid engraftment. Conversely, the patient who underwent myeloablative conditioning (P8) achieved 100% donor chimerism in both the lymphoid and myeloid compartments. The patients’ detailed transplant outcomes and immune reconstitution data are summarized in **Table 2**.

**Table 2:** HSCT characteristics and outcomes for transplanted *ZAP70* patients.

P	Age at 1st HSCT (months)	Second HSCT (months)	Stem Cell Source	Donor & HLA Match	Conditioning/Regimen	GVHD Prophylaxis	CD34 dose/kg (10^6^/kg)	STR Myeloid (M), Lymphoid (L)	Engraftment (Platelet/Neutrophil) Days	CD8 (mm³) (Normal range)	PHA patient/control (%)	Complications, Infections, Autoimmunity	Life Status, Cause of Death	Years Post-HSCT
P1	24	No	BM	Father, FMD	No	CSA	9.08	M:0%,L:50%	–	208 (300 – 1000)	31	–	Alive, healthy	15
P2	84	No	BM	Mother, FMD	No	CSA	6.56	M:0%,L:76%	–	460 (300 – 1000)	7	Vitiligo	Alive, healthy	11
P3	72	No	BM	Father, FMD	No	CSA	3.73	M:0%, L45%	–	272 (300 – 1000)	ND	–	Alive, FTT	11
P4	3	Yes, at 48 months	BM	Mother, FMD for GVHD, Haploidentical for rejection	1^st^: RIC: Fludarabine, Melphalan, ATG 2^nd^: boost	CSA	8.15	M:0%,L:92%	–	129 (300 – 1100)	101	AIHA, intermittent neutropenia, CMV	alive, healthy	10
P5	7	No	BM	Sibling, FMD	No	–	10	M:0%,L:38%	–	106 (400 – 1200)	ND	GVHD Skin grade 2	Alive, recurrent URTI, bronchiectasis, onychomycosis	4
P6	7	No	BM	Sibling, FMD	No	–	10	M:0%,L:52%	–	771 (400 – 1200)	ND	–	Alive, healthy	3
P7	8	No	BM	Sibling, FMD	No	–	10	M:0%, L:59%	–	677 (500 – 1500)	ND	–	Alive, healthy	1.6
P8	4	Yes, at 36 months	BM	Father, FMD	1^st:^ No 2^nd^: MAC: Busulfan, Fludabine, ATG	Tacrolimus, Methotrixate	4.38	M:100%,L:95%	21/23	248 (400 – 1200)	ND	Toxic erythema of chemotherapy	Alive, healthy	1
P9	5	No	BM	Father, One antigen mismatch	No	CSA,MMF	5	–	–	–		–	Dead, Septicemia	–
P10	3	No	BM	Sibling, One antigen mismatch	Yes/MAC: Busulfan, Cyclophosphamide	CSA,Methotrexate	9.83	–	16/30	–		–	Dead, ARDS	–
P11	22	No	Cord blood	Cord, Unrelated donor	Yes/RIC: Fludarabine, Melphalan, ATG	CSA, MMF, Methylprednisolone	TNC: 5.15 × 108/kg	–	Not engrafted/33	–		GVHD skin and gut grade 2, VOD	Dead, ARDS	–

HSCT, Hematopoietic Stem Cell Transplantation; BM, Bone marrow; FMD, Family matched donor, CSA, Cyclosporine; MMF, Mycophenolate mofetil; RIC, Reduced intensity conditioning; MAC, Myeloablative conditioning; ATG, Anti-thymocyte globulin; TNC, Total nucleated cells; ND, not done; AIHA, Autoimmune hemolytic anemia; CMV, Cytomegalovirus; VOD, Veno-Occlusive Disease.

### Immune reconstitution

At last follow-up, eight surviving patients showed evidence of immune reconstitution after HSCT, as demonstrated by both clinical improvement and immunological recovery. Clinically, these patients showed resolution of recurrent infections, clearance of viral reactivations, and were able to discontinue antimicrobial prophylaxis. Immunologically, most achieved normalization of CD3^+^ T-cell counts with partial recovery of CD8^+^ T-cell counts (range, 106–771 cells/mm3), restoration of mitogen-induced T-cell proliferation among tested patients and stable donor lymphoid chimerism. Seven patients discontinued IVIG replacement and were able to maintain normal serum IgG, IgA, IgM) and mounting protective vaccine-specific antibody responses. One patient (P2) demonstrated partial functional reconstitution, with mitogen-induced T-cell proliferation remaining suboptimal (7% of control), despite achieving adequate CD8^+^ T-cell counts and remaining clinically well without infections (**Table 2**).

## Discussion

*ZAP70* deficiency is a rare autosomal recessive form of combined immunodeficiency characterized by disrupted TCR signaling, primarily affecting CD8+ T-cell development and function. The disease is currently recognized as a distinct entity within the spectrum of SCID, featuring a highly variable phenotype ranging from classical early-onset SCID to late-onset immune dysregulation. Clinical experience remains limited because of its rarity, and treatment practices, especially regarding HSCT, are evolving. This study presents the largest single-center cohort of genetically confirmed *ZAP70* deficiency, offering valuable insights into the clinical, immunological, and genetic characteristics of the disease, as well as HSCT outcomes and immune reconstitution.

The ZAP70 gene encodes a tyrosine kinase critical for TCR signaling. Previous reports described 32 different mutations in 49 patients, the majority being homozygous or compound heterozygous, scattered across the gene without an apparent genotype–phenotype correlation (23). In our cohort of 13 patients, five distinct homozygous mutations were identified. Remarkably, the G536S variant was found in eight individuals, suggesting a founder effect in our regional population. Although G536S was associated with both classical SCID features and atypical presentations in our cohort, this mirrors broader observations that clinical severity might not be directly predicted by genotype. The remaining mutations were unique to individual families.

A 2020 systematic review of 49 *ZAP70*-deficient patients reported a wide spectrum of clinical manifestations, including recurrent respiratory infections (81.8%), cutaneous involvement (57.9%), lymphoproliferation (32.4%), autoimmunity (19.4%), enteropathy (18.4%), and malignancy (8.1%). Infections were predominantly viral (CMV, EBV, varicella, rotavirus), but bacterial, fungal, and mycobacterial pathogens were also detected (e.g., BCGitis, *Pneumocystis jirovecii*). Autoimmune complications were also documented, including cytopenia, nephritis, and enteropathy (23). The clinical presentation of our cohort overlaps with previously reported cases, but novel features were also revealed. Although recurrent respiratory infections, chronic diarrhea, failure to thrive, and dermatitis were common presenting signs, we also observed allergic phenotypes, including food allergy and allergic rhinitis in some patients. These findings, which were not widely reported in previous cohorts, might reflect underlying Th2 skewing or dysregulated immune responses in partially functional T-cells. Additionally, autoimmune phenomena, including cytopenia and nephrotic syndrome, were detected in three unrelated patients carrying the same mutation, raising questions about immune tolerance defects related to specific variants or tribal gene pools. The median age at genetic diagnosis was 4 months, earlier than the reported median of 7.5 months.

Immunologically, CD8^+^ lymphopenia remains the hallmark of *ZAP70* deficiency, being reported in more than 95% of cases (23). Similarly, 11 of 13 patients in our study exhibited markedly reduced CD8^+^ T-cell counts. However, two patients (P2 and P3), who presented later in life at the ages of 5 and 6 years respectively, had normal-for-age CD8^+^ counts but demonstrated profoundly impaired T-cell proliferation in response to mitogen stimulation. Notably, both patients carried the G536S mutation, which was also identified in other individuals with classical early-onset disease and severe CD8^+^ lymphopenia, underscoring the phenotypic variability associated with this genotype. A similar *ZAP70*-deficient patient with a high CD8^+^ count was recently reported, albeit a different gene variant (24). These observations highlight the critical importance of integrating immunophenotyping with functional assays, such as lymphocyte proliferation testing, and utilizing unbiased genetic approaches, including primary immunodeficiency gene panels or whole-exome sequencing. for timely and accurate diagnosis, particularly in atypical or late-onset cases.

Immunoglobulin levels in our cohort were generally normal, consistent with literature reports, but specific antibody responses to vaccines were often deficient. This functional humoral impairment underscores the importance of early Ig replacement and infection prophylaxis in disease management, particularly before definitive therapy with HSCT. As expected, mitogen-induced lymphocyte proliferation (e.g., to PHA) was profoundly reduced in most patients, highlighting the utility of this test for assessing T-cell function in suspected cases.

The pathogenesis of autoimmunity in *ZAP70* deficiency is increasingly recognized and likely multifactorial. Animal models and human studies suggest defective regulatory T-cell generation and function, reduced CTLA-4 and TGF-β expression, and poor IL-10 production attributable to impaired Th2 differentiation (15). Among our cohort, autoimmune cytopenia and nephrotic syndrome were observed in two unrelated patients from the same tribe, both carrying the same mutation. As reported in previous studies, autoimmune manifestations can occur in patients with *ZAP70* deficiency. For example, one case report described a 3-year-old child presenting with autoimmune thrombocytopenia and ulcerative colitis, illustrating the potential for significant immune dysregulation in affected individuals (25). Although autoimmunity might be underreported because of early mortality, its presence in our cohort and other studies indicates the need for ongoing surveillance and potentially represents a clinical clue in atypical or late-onset presentations.

HSCT remains the only curative treatment for *ZAP70* deficiency. Early transplantation, ideally before the onset of significant infections or organ damage, offers the best chance for immune reconstitution and long-term survival. However, because of diagnostic delays and limited donor availability, many patients undergo HSCT under suboptimal conditions. To date, successful HSCT has been reported in approximately 25 patients, with reported survival rates exceeding 90%, although detailed transplants procedure and long-term outcome data are not available for most cases (23). Cuvelier et al. reported the long-term outcomes of eight patients with *ZAP70* deficiency who underwent HSCT with a median follow-up of 13.5 years. Three of these patients received unconditioned bone marrow transplants from HLA-matched sibling donors. These individuals developed persistent mixed T-cell chimerism but exhibited poor or absent engraftment in the B-cell and myeloid lineages. Remarkably, despite the absence of full multilineage engraftment, all three achieved normal serum Ig levels, mounted protective antibody responses following post-transplant vaccinations, and successfully discontinued Ig replacement therapy. The remaining five patients received myeloablative conditioning, including three patients who received T-cell–depleted haploidentical grafts and two patients who received cord blood transplants from unrelated donors, and demonstrated complete donor chimerism across the T-cell, B-cell, and myeloid compartments. Following HSCT, CD8^+^ T-cell counts were normal in five patients, elevated in one patient, and reduced in two patients. Importantly, all eight patients exhibited normal lymphocyte proliferation in response to PHA stimulation, and seven no longer required Ig replacement (26).

Our experience highlights the complexity of selecting the optimal HSCT strategy for ZAP70 deficiency. Outcomes were strongly influenced by donor type, conditioning intensity, and the patient’s clinical status at the time of transplant. HLA-matched sibling donors without conditioning achieved survival with partial lymphoid chimerism, which was sufficient to restore T-cell function and protect against infections. However, these patients generally lacked myeloid engraftment, raising questions about long-term durability. In contrast, haploidentical or mismatched related donor transplants were associated with higher transplant-related mortality, particularly in patients with active infections at the time of HSCT. Myeloablative conditioning regimens, while more toxic, resulted in full donor chimerism across hematopoietic lineages and more complete immune reconstitution, as illustrated by patient P8 who required a second transplant. Importantly, conditioning must be tailored to the individual: in patients with ongoing infections or organ dysfunction, unconditioned or reduced-intensity approaches may serve as lifesaving options, particularly with T-cell–replete grafts or HLA-matched donors. Overall, our findings suggest that while unconditioned or reduced-intensity regimens have a role in selected high-risk patients, myeloablative conditioning remains the most reliable strategy for achieving durable multilineage engraftment when clinically feasible.

This study has several limitations. First, the number of transplanted patients was small (n=11), reflecting the rarity of *ZAP70* deficiency. Consequently, formal survival analyses (e.g., Kaplan–Meier curves) or subgroup comparisons were not performed, as the results would be underpowered and confounded by the heterogeneity of our cohort. Patients varied considerably in age at HSCT, donor type, conditioning intensity, and clinical status at the time of transplant, including the presence of active infections and organ dysfunction. These differences would substantially affect outcomes and could render statistical comparisons misleading. Instead, we chose a descriptive approach that highlights clinically meaningful patterns without overstating conclusions. Second, all patients were treated at a single tertiary referral center in a highly consanguineous population with a regional founder mutation effect. While this provides valuable insight into genotype–phenotype correlation, it may limit the generalizability of our findings to more genetically diverse populations. Collaborative multicenter studies will therefore be essential to validate these observations and refine transplant strategies for ZAP70 deficiency.

## Conclusion

*ZAP70* deficiency presents with a broad spectrum of clinical and immunological features, including atypical phenotypes such as normal CD8^+^ counts and allergic or autoimmune manifestations. High clinical suspicion, particularly in consanguineous populations with recurrent infections and CD8^+^ lymphopenia or abnormal lymphocyte function, is critical for early diagnosis. HSCT remains the definitive therapy. Myeloablative conditioning is associated with more complete immune reconstitution, but unconditioned transplants might be suitable for patients with contraindications to intensive regimens. Given the rarity of this disease, collaborative multicenter efforts are needed to refine transplant protocols and further characterize long-term outcomes.

## List of abbreviations

SCID, Severe combined immunodeficiency disease; HSCT, Hematopoietic stem cell transplantation; HLA, Human Leukocyte Antigen; GVHD, Graft-versus-host disease; TCR, T-cell receptor; KFSHRC, King Faisal Specialist Hospital and Research Center; PHA, Phytohemagglutinin; BCG, Bacillus Calmette–Guérin; ATG, Antithymocyte globulin; CMV, Cytomegalovirus; EBV, Epstein–Barr virus; IVIG, Intravenous immunoglobulin; AIHA, Autoimmune hemolytic anemia; Ig, Immunoglobulin; PTK, Protein tyrosine kinase; ZAP70, ζ-chain-associated protein kinase of 70 kDa; CSA, Cyclosporine; ANC, Absolute neutrophil count; NK, natural killer; VOD, Veno-Occlusive Disease; LAP, Lymphadenopathy.

## Data Availability

The raw data supporting the conclusions of this article will be made available by the authors, without undue reservation.

## References

[1] Fischer A Cavazzana-Calvo M De Saint Basile G DeVillartay JP Di Santo JP Hivroz C. Naturally occurring primary deficiencies of the immune system. Annu Rev Immunol. (1997) 15:93–124. doi: 10.1146/annurev.immunol.15.1.93, PMID: 9143683

[2] WHO Report. Primary immunodeficiency diseases report of an IUIS scientific committee. Clin Exp Immunol. (1999) 118:1–28. doi: 10.1046/j.1365-2249.1999.00109.x, PMID: 10540200 PMC1905383

[3] Villa A. V (D) J recombination defects in lymphocytes due to RAG mutations: severe immunodeficiency with a sp*ectrum of clinical presentations* . Blood. (2001) 97:81–8. doi: 10.1182/blood.V97.1.81, PMID: 11133745

[4] Bousfiha AA Jeddane L Moundir A Poli MC Aksentijevich I Cunningham-Rundles C. The 2024 update of IUIS phenotypic classification of human inborn errors of immunity. J Hum Immunol. (2025) 1(1):e20250002. doi: 10.70962/jhi.20250002, PMID: 36198931

[5] de Saint Basile G Geissmann F Flori E Uring-Lambert B Soudais C Cavazzana-Calvo M. Severe combined immunodeficiency caused by deficiency in either the delta or the epsilon subunit of CD3. J Clin Invest. (2004) 114(10):1512–7. doi: 10.1172/JCI22588, PMID: 15546002 PMC525745

[6] Giblett ER Anderson JE Cohen F Pollara B Meuwissen HJ. Adenosine-deaminase deficiency in two patients with severely impaired cellular immunity. Lancet. (1972) 2(7786):1067–9. doi: 10.1016/S0140-6736(72)92345-8, PMID: 4117384

[7] Kung C Pingel JT Heikinheimo M Klemola T Varkila K Yoo LI. Mutations in the tyrosine phosphatase CD45 gene in a child with severe combined immunodeficiency disease. Nat Med. (2000) 6(3):343–5. doi: 10.1038/73208, PMID: 10700239

[8] Macchi P Villa A Giliani S Sacco MG Frattini A Porta F. Mutations of Jak-3 gene in patients with autosomal severe combined immune deficiency (SCID). Nature. (1995) 377(6544):65–8. doi: 10.1038/377065a0, PMID: 7659163

[9] Noguchi M. Interleukin-2 receptor gamma chain mutation results in X-linked severe combined immunodeficiency in humans. Cell. (1993) 73:147–57. doi: 10.1016/0092-8674(93)90167-O, PMID: 8462096

[10] Puel A Ziegler SF Buckley RH Leonard WJ. Defective IL7R expression in T(-)B(+)NK(+) severe combined immunodeficiency. Nat Genet. (1998) 20(4):394–7. doi: 10.1038/3877, PMID: 9843216

[11] Tchilian EZ Wallace DL Wells RS Flower DR Morgan G Beverley PC. A deletion in the gene encoding the CD45 antigen in a patient with SCID. J Immunol. (2001) 166(2):1308–13. doi: 10.4049/jimmunol.166.2.1308, PMID: 11145714

[12] Wang H Kadlecek TA Au-Yeung BB Goodfellow HE Hsu LY Freedman TS. ZAP-70: an essential kinase in T-cell signaling . Cold Spring Harb Perspect Biol. (2010) 2(5):a002279–9. doi: 10.1101/cshperspect.a002279, PMID: 20452964 PMC2857167

[13] Sharifinejad N Jamee M Zaki-Dizaji M Lo B Shaghaghi M Mohammadi H. Clinical, immunological, and genetic features in 49 patients with ZAP-70 deficiency: a systematic review. Front Immunol. (2020) 11:831. doi: 10.3389/fimmu.2020.00831, PMID: 32431715 PMC7214800

[14] Huber RG Fan H Bond J. The structural basis for activation and inhibition of ZAP-70 kinase domain. PLoS Comput Biol. (2015) 11:e1004560. doi: 10.1371/journal.pcbi.1004560, PMID: 26473606 PMC4608720

[15] Roifman CM Dadi H Somech R Nahum A Sharfe N. Characterization of zeta-associated protein, 70 kd (ZAP70)-deficient human lymphocytes. J Allergy Clin Immunol. (2010) 126(16):1226–33.e1. doi: 10.1016/j.jaci.2010.07.029, PMID: 20864151

[16] Roifman CM. A mutation in zap-70 protein tyrosine kinase results in a selective immunodeficiency. J Clin Immunol. (1995) 15:S52–62. doi: 10.1007/BF01540894, PMID: 8613493

[17] Manjunatha BS Das N Naik S Gowramma R. Trabecular variant of juvenile aggressive ossifying fibroma of anterior mandible. Pediatr Rep. (2012) 4(2):e24. doi: 10.4081/pr.2012.e24, PMID: 22803002 PMC3395982

[18] Gohar AA Maatooq TG Gadara RS Aboelmaaty SW El-Shazly MA. Molluscicidal activity of the methanol extract of callistemon viminalis (Sol. ex Gaertner) G.Don ex loudon fruits, bark and leaves against Biomphalaria alexandrina Snails. Iran J Pharm Res. (2014) 13(2):505–14. Available online at: https://pubmed.ncbi.nlm.nih.gov/25237345/, PMID: 25237345 PMC4157025

[19] Macnamara B Palucka KA Porwit-MacDonald A. Balance between proliferation and apoptosis in leukemic cell lines resistant to cytostatics. Leuk Lymph. (1999) 36:179–89. doi: 10.3109/10428199909145962, PMID: 10613463

[20] Roep BO Kallan AA Duinkerken G Arden SD Hutton JC Bruining GJ. T-cell reactivity to beta-cell membrane antigens associated with beta-cell destruction in IDDM. Diabetes. (1995) 44(3):278–83. doi: 10.2337/diab.44.3.278, PMID: 7883114

[21] Routier FH Hounsell EF Rudd PM Takahashi N Bond A Hay FC. Quantitation of the oligosaccharides of human serum igg from patients with rheumatoid arthritis: a critical evaluation of different methods. J Immunol Methods. (1998) 213(2):113–30. doi: 10.1016/S0022-1759(98)00032-5, PMID: 9692845

[22] Al-Mousa H Abouelhoda M Monies DM Al-Tassan N Al-Ghonaium A Al-Saud B. Unbiased targeted next-generation sequencing molecular approach for primary immunodeficiency diseases. J Allergy Clin Immunol. (2016) 137(6):1780–7. doi: 10.1016/j.jaci.2015.12.1310, PMID: 26915675

[23] Sharifinejad N Jamee M Zaki-Dizaji M Lo B Shaghaghi M Mohammadi H. Clinical, immunological, and genetic features in 49 patients with ZAP-70 deficiency. Front Immunol. (2020) 11:831. doi: 10.3389/fimmu.2020.00831, PMID: 32431715 PMC7214800

[24] Aluri J Desai M Gupta M Dalvi A Terance A Rosenzweig SD. Clinical, immunological, and molecular findings In 57 patients with severe combined immunodeficiency (SCID) from India. Front Immunol. (2019) 10:23. doi: 10.3389/fimmu.2019.00023, PMID: 30778343 PMC6369708

[25] Harville T. 829 Elevated TNF-α associated with neutropenia in combined immunodeficiency (CID). J Allergy Clin Immunol. (1996) 829:390. doi: 10.1016/S0091-6749(96)81047-5

[26] Cuvelier GD Rubin TS Wall DA Schroeder ML. Long-Term outcomes of hematopoietic stem cell transplantation for ZAP70 deficiency. J Clin Immunol. (2016) 36(7):713–24. doi: 10.1007/s10875-016-0316-z, PMID: 27438785

